# Supplementary material for: Complex tsunamigenic near-trench seafloor deformation during the 2011 Tohoku–Oki earthquake
Source: Nat Commun. 2023 Jun 5;14:3260. doi: 10.1038/s41467-023-38970-z (PMC10241807; doi:10.1038/s41467-023-38970-z)
Supplement: Supplementary file 1 — Supplementary Information [file 41467_2023_38970_MOESM1_ESM.pdf]

Supplementary Information for

Complex near-trench coseismic deformation in the 2011 Tohoku–Oki earthquake: insight from improved-resolution differential bathymetry

This file includes:

Supplementary Figures 1 to 5

Supplementary Tables 1

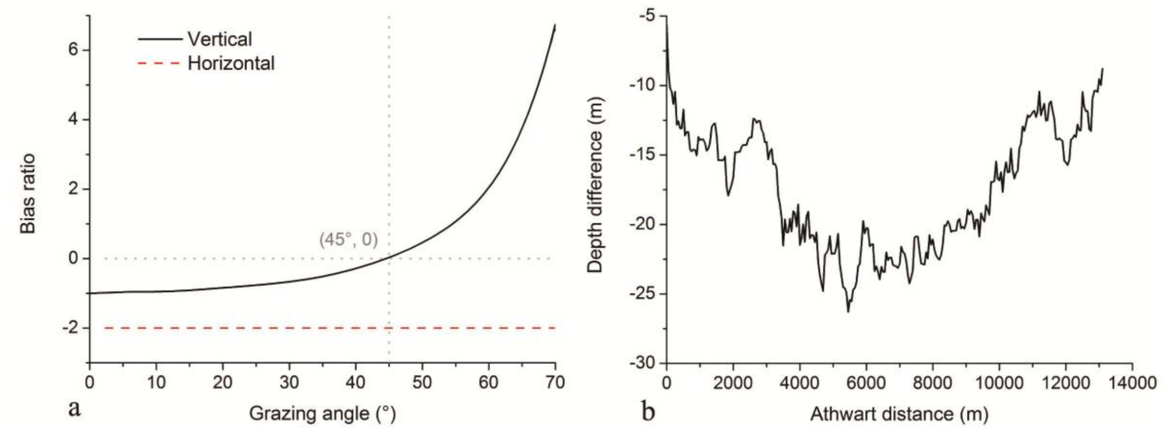

Supplementary Fig. 1 Effect of inaccurate SVP information. (a) Ratio between the relative vertical and horizontal error in soundings and the relative error in SVP corresponding to different grazing angles. (b) A typical athwart profile of depth difference values in track 1.

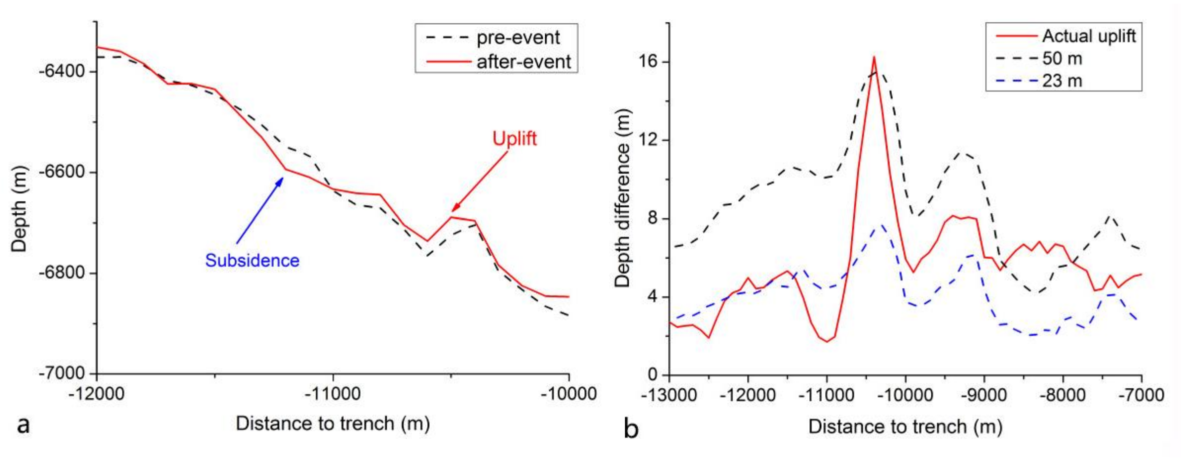

Supplementary Fig. 2 Local depth variation near the anomalous seafloor uplift peak of track 1. (a) Local seafloor bathymetry profiles before and after the earthquake. (b) Comparison of the actual and the synthetic differential bathymetry profiles. The red curve denotes the actual differential bathymetry profile along the dip-slip direction. The blue and red dashed curves denote the synthetic differential bathymetry profile associated with horizontal displacements of 23 m (the bathymetry matching result) and 50 m, respectively.

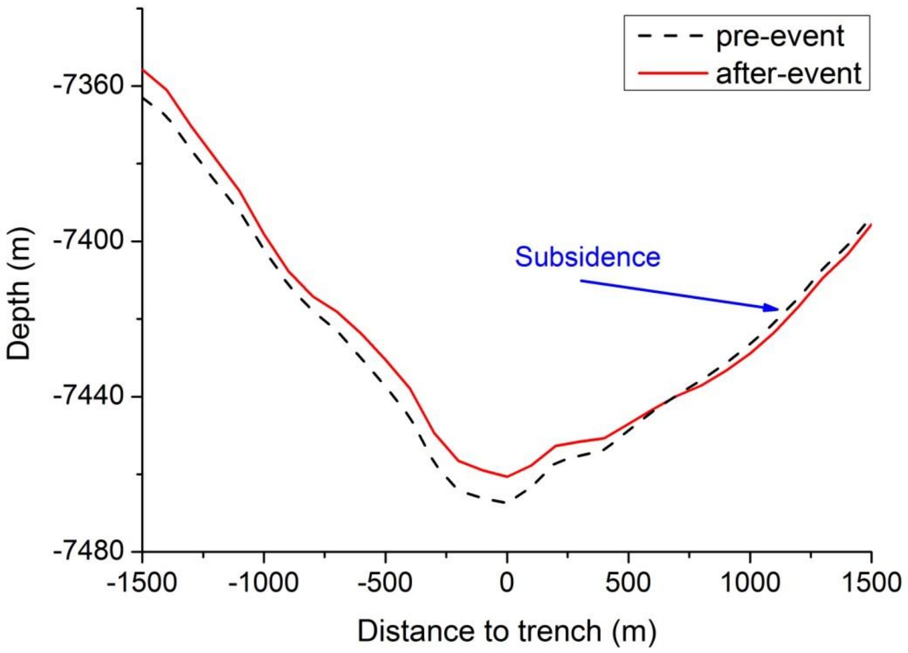

Supplementary Fig. 3 Local depth variation proximal to the trench for track 1. The black dashed and red solid curves denote the bathymetry profile before and after the earthquake.

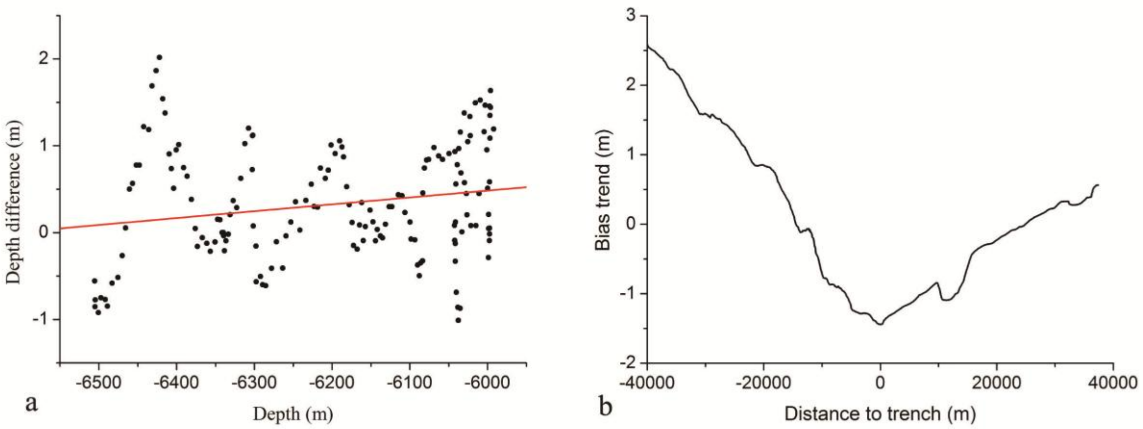

Supplementary Fig. 4 Correction of the depth-dependent bias in vertical depth differences of track 1. (a) Estimation of the depth-dependent bias term. The black dots denote the differential bathymetry profile associated with the outer-rise region where coseismic influence is expected to be negligible. The red line denotes the best-fit trend. (b) The estimated bias trend across the track.

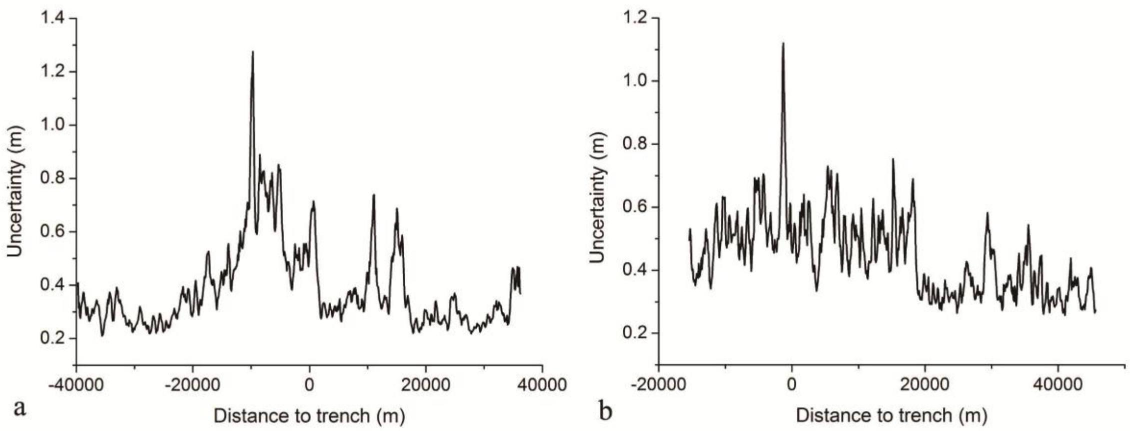

Supplementary Fig. 5 Vertical uncertainty (1σ) associated with the depth difference profile. (a) Track 1. (b) Track 2.

Supplementary Table 1 Cruise information for the two tracks

Track number	Before-event line number	Post-event line number	Before-event cruise name	Post-event cruise name
1	20010713	20120603	MR01-K03	YK12-08
2	20101116	20110629	KR10-12	YK11-E05
